# Healthcare Research and Analytics Data Infrastructure Solution: A Data Warehouse for Health Services Research

**DOI:** 10.2196/18579

**Published:** 2020-06-04

**Authors:** Bunyamin Ozaydin, Ferhat Zengul, Nurettin Oner, Sue S Feldman

**Affiliations:** 1 University of Alabama at Birmingham Birmingham, AL United States

**Keywords:** health services research, data warehousing, iterative process model, systems analysis and design, data integration

## Abstract

**Background:**

Health services researchers spend a substantial amount of time performing integration, cleansing, interpretation, and aggregation of raw data from multiple public or private data sources. Often, each researcher (or someone in their team) duplicates this effort for their own project, facing the same challenges and experiencing the same pitfalls discovered by those before them.

**Objective:**

This paper described a design process for creating a data warehouse that includes the most frequently used databases in health services research.

**Methods:**

The design is based on a conceptual iterative process model framework that utilizes the sociotechnical systems theory approach and includes the capacity for subsequent updates of the existing data sources and the addition of new ones. We introduce the theory and the framework and then explain how they are used to inform the methodology of this study.

**Results:**

The application of the iterative process model to the design research process of problem identification and solution design for the Healthcare Research and Analytics Data Infrastructure Solution (HRADIS) is described. Each phase of the iterative model produced end products to inform the implementation of HRADIS. The analysis phase produced the problem statement and requirements documents. The projection phase produced a list of tasks and goals for the *ideal* system. Finally, the synthesis phase provided the process for a plan to implement HRADIS. HRADIS structures and integrates data dictionaries provided by the data sources, allowing the creation of dimensions and measures for a multidimensional business intelligence system. We discuss how HRADIS is complemented with a set of data mining, analytics, and visualization tools to enable researchers to more efficiently apply multiple methods to a given research project. HRADIS also includes a built-in security and account management framework for data governance purposes to ensure customized authorization depending on user roles and parts of the data the roles are authorized to access.

**Conclusions:**

To address existing inefficiencies during the obtaining, extracting, preprocessing, cleansing, and filtering stages of data processing in health services research, we envision HRADIS as a full-service data warehouse integrating frequently used data sources, processes, and methods along with a variety of data analytics and visualization tools. This paper presents the application of the iterative process model to build such a solution. It also includes a discussion on several prominent issues, lessons learned, reflections and recommendations, and future considerations, as this model was applied.

## Introduction

There are a variety of data sources most frequently used for health services research, a multidisciplinary research field that investigates the implications of factors such as social determinants, organizational structures and processes, technologies, financing and reimbursement, individual choices and behaviors on the access and quality of health care delivery, and overall health and well-being of individuals [1]. Most of the data sources for health services research are provided by the Centers for Medicare and Medicaid Services (CMS); however, there are also data sources provided by other government agencies and nonprofit or for-profit data providers. Health services researchers, especially those using secondary data, can expand their research analytics by using merged datasets for health services research. In the absence of a single data warehouse from which to retrieve and analyze data from previously disparate datasets, health services researchers are forced to perform separate and often redundant data-related tasks on each individual dataset. Anecdotal reports suggest that researchers spend as much as 60% of their time on data preparation. At best, we can describe the current data-related processes as inefficient, costly, time-consuming, and cumbersome [2]. Moreover, the current uncoordinated and isolated efforts on these disparate datasets can be wasteful as they may generate research findings that are not reproducible or sometimes misleading because of the unaddressed inherent problems within these datasets. Furthermore, without the needed information technology (IT) infrastructure, analytics, and data visualization tools, the potential of the ever-growing health-related big data accumulated in these disparate datasets would still be untapped [3]. Therefore, there is a need for a cyberinfrastructure that integrates these disparate databases in a secure and consistent manner and provides the necessary analytics and visualization tools.

### Background: Systems Around Health Data

**Figure 1:**
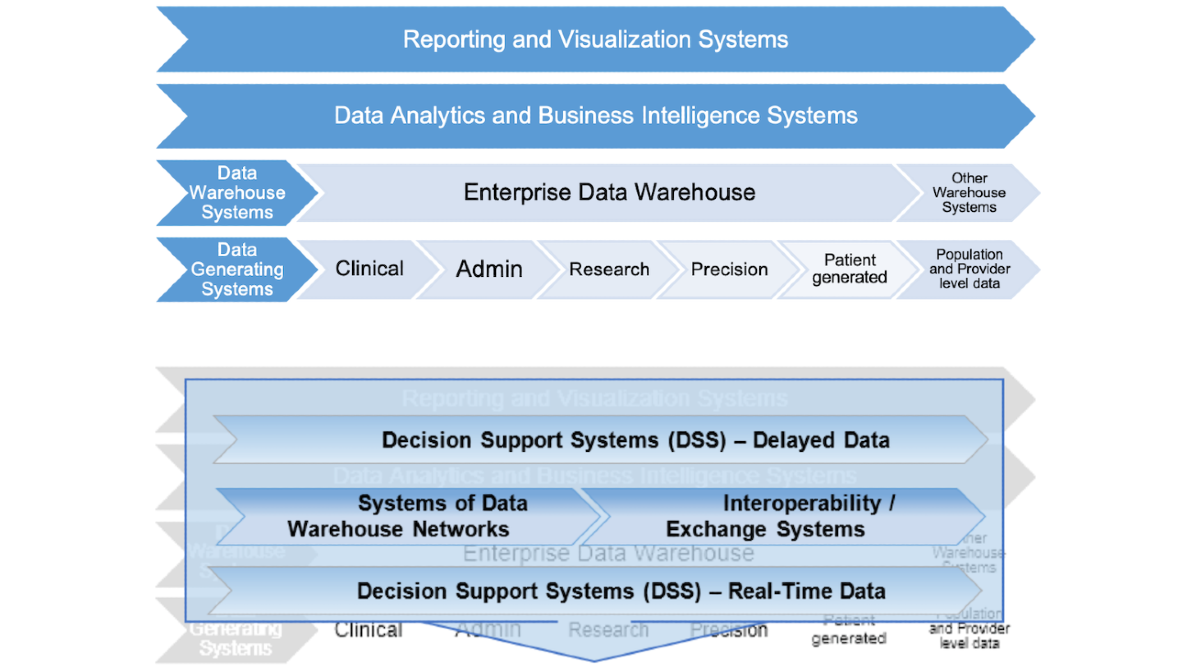
Systems that deal with health-related data: First layer (top), second layer (bottom).

In its life cycle, health-related data mainly move through four types of systems, as indicated in the top part of Figure 1 (adapted from the study by Ozaydin et al [4]). Patient-level data are usually generated in one of the operational systems that fall into categories of clinical, administrative, research, and precision medicine systems and systems that manage medical devices that patients use. The clinical systems include everything that is part of the electronic health record (EHR) and systems dealing with laboratories, imaging, physician notes, medications, histories, procedures, and diagnoses, regardless of whether or not they are part of the EHR. Administrative systems include admittance-discharge-transfer; billing, scheduling, and claims systems; as well as systems that are not specific to health care, such as systems that manage human resources and payroll. The research-related health data are generated by the systems for clinical research, clinical trials, and various registries. Furthermore, there are systems generating precision medicine data, such as genomics, phonemics, and microbiome, and systems where patient-generated data are generated, such as mobile health and telehealth systems, internet-of-things, and other data-generating medical devices, social media, and patient portals. After being created in one of the data-generating systems, the patient-level data are usually aggregated at an institutional enterprise data warehouse system. These data warehouses usually serve as the infrastructure on which institutional data analytics and business intelligence (BI) systems—based on which reporting and visualization systems, such as dashboards—run [4]. There are also other data warehouse systems outside of individual institutions, such as systems used for public health purposes [5,6].

As indicated in the bottom part of Figure 1, there are also second-layer systems that operate between the systems shown in the top part of Figure 1. The second-layer systems include decision support systems, systems that provide interoperability between the first-layer systems (ie, interface engines), health information exchanges, and networks of institutional data warehouses. These second-layer systems are closer to data-generating systems if they are required to use real-time data. In the absence of the use of real-time data, these systems rely on delayed data provided by the data warehouse systems. As decision support systems mature, there are increasing expectations to provide their results back to the data-generating systems as close to real time as possible. To date, several data warehouse networks, such as the networks of informatics for integrating biology and the bedside (i2b2) systems called the shared health research information network, have been developed as data warehouse networks to integrate clinical and administrative data extracted from various systems of health care entities.

### Need for the Healthcare Research and Analytics Data Infrastructure Solution

The systems described so far are mostly geared toward integrating patient-level electronic health, billing, and other administrative data to be used for clinical and translational research [2], without much focus on the organizational-level data. The first-layer systems also include systems that generate population- and provider-level data and data warehouse systems for them as indicated on the right-hand side of Figure 1. The population-level warehouse systems focus on epidemiological systems, systems managing national and regional indexes and surveys, and systems managing the Centers for Disease Control and Prevention databases. The provider-level warehouse systems focus on systems that manage data for health services administration, such as quality measures, satisfaction scores, inspections, financial performance, and services offered.

In addition to data warehouse networks for patient-level data, there have also been attempts to create integrated data repositories to include certain portions of the selected data sources for health services research for various purposes (ie, Research Data Assistance Center [ResDAC] [7] and Wharton research data services [WRDS] [8]). However, we could not find evidence of any mature platform that integrates all of the targeted data sources mentioned in Textbox 1 or of any effort to create such a platform in the literature. A majority of health services data continue to aggregate and evolve as isolated silos within various governmental or nongovernmental entities [2], with research efforts to interpret the data also operating in silos. In an era where the generation of data surpasses the efforts to extract meaning out of it, these uncoordinated silos of research efforts delay the necessary improvements in much-needed research efficiency. Therefore, enhancing health services research efficiency necessitates a platform that has the potential to integrate the disparate silos of datasets and research efforts.

**Data sources included in the first phase of the Healthcare Research and Analytics Data Infrastructure Solution.**

- Centers for Medicare and Medicaid Services (CMS) Medicare cost reports
- CMS impact and final rule files
- Datasets from CMS Hospital Compare, including Hospital Consumer Assessment of Healthcare Providers and Systems
- Area health resources files
- American Hospital Association (AHA) annual survey
- AHA health information technology supplement
- Dartmouth Atlas
- Bureau of Labor Statistics

## Methods

This section introduces data sources and architecture for the Healthcare Research and Analytics Data Infrastructure Solution (HRADIS) platform and the theoretical orientation and explains how the theoretical background is used to inform the methodology for this study.

### Healthcare Research and Analytics Data Infrastructure Solution Data Sources and Architecture

**Figure 2:**
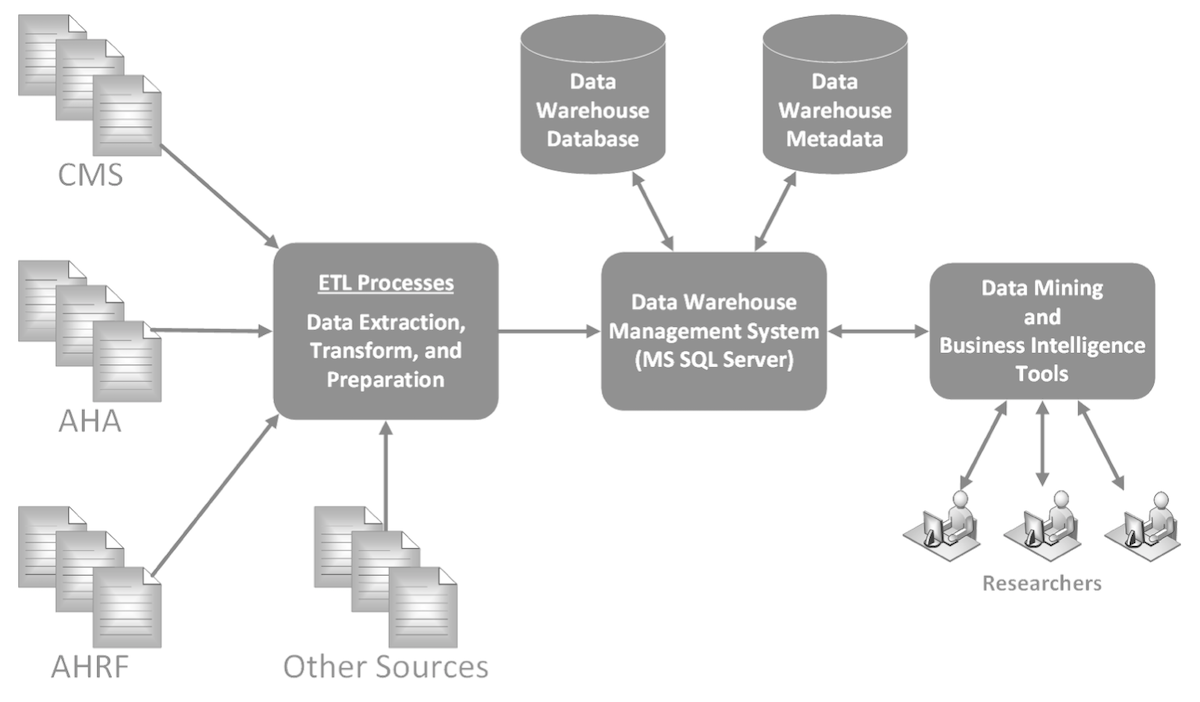
The healthcare research and analytics data infrastructure solution architecture. CMS: Centers for Medicare and Medicaid Services; AHA: American Hospital Association; AHRF: area health resources files.

To address the aforementioned need, this project aimed to generate a cyberinfrastructure by initially creating a data warehouse using the Microsoft SQL Server platform to integrate these frequently used health services data sources in a reliable, secure, and consistent manner and then to build a BI system that includes tools for data mining, analytics, and visualization, as depicted in Figure 2 (adapted from Kroenke and Auer [9]). The elements of HRADIS include data; metadata; procedures and applications of the data and metadata; other data tools; and users, groups, and data access policies. As shown in Figure 2, there are several different areas of data interaction. First, the data interact with the ETL (extract, transform, load) processes where data are prepared for storage in the data warehouse. Next, the data warehouse management system stores the data and metadata and handles data interaction between various other system tools and the stored data. Finally, the health services researchers interact with a graphical user interface to access the data through data mining and BI tools. The first phase of HRADIS hosts data from the data sources listed in Textbox 1.

Some of the data sources listed in Textbox 1 have been made available through several research data centers such as ResDAC at the University of Minnesota, WRDS at the University of Pennsylvania, and the National Bureau of Economic Research (NBER) [10]. However, these research data centers do not include most of the data sources listed in Textbox 1. They primarily provide training and technical assistance on specific data sources such as CMS Medicare and Medicaid data in the case of ResDAC, focus on nonhealth care areas such as finance and business in the case of WRDS or improve the accessibility of existing data sources such as CMS Medicare cost reports (MCR) in the case of NBER. There are also commercial data centers, which provide reports on quality, finance, and inpatient and outpatient outcomes, for individual hospitals such as the American hospital directory [11] or hospital profiles such as Hospital-data [12]. However, these commercial data centers are not as comprehensive as, and some lack research focus when compared with HRADIS.

HRADIS is designed to be sustainable and scalable so that the inclusion of new data sources and updates of existing ones is efficient. This allows health services researchers to apply their models to updated data or data from new sources without having to merge new data to their research datasets.

A challenge exists in interpreting complex data dictionaries, layouts, and other metadata elements that accompany raw data to be able to identify and reliably extract parameters of interest for a given research project. To address this issue, HRADIS integrates metadata and ETL processes that utilize it to identify and extract parameters of interest based on how the parameters are defined by the metadata, rather than keeping metadata in a separate file repository or in an accompanying document warehouse [13]. To accomplish this, the source data are put through an initial phase of ETL tasks to populate database tables created based on the relational HRADIS data model in the entity relationship diagram format. Furthermore, HRADIS includes a second phase of ETL tasks to populate dimensions and measures that are created for the most frequently used parameters for more efficient performance of data mining, analytics, and visualization tasks based on the multidimensional HRADIS data model in a star schema diagram format. In other words, HRADIS benefits the advantages of both relational and dimensional models and their diagrammatical representations, as described in studies by Corral et al [14] and Schuff et al [15].

Currently, we have loaded MCR, AHA, Hospital Consumer Assessment of Healthcare Providers and Systems (HCAHPS), Dartmouth Atlas, Bureau of Labor Statistics (BLS), and parts of Hospital Compare datasets with HRADIS and have begun data extraction from multiple data sources for pilot projects. Although most of the data incorporated into the system come from publicly accessible sources, some of the data are restricted. As we populate HRADIS with data, we are also implementing the security and data governance components of the system. Security and data governance are important for users to access only the parts of the data for which they are authorized.

### Sociotechnical Systems Theory

Although cyberinfrastructures can be designed and developed in a system-centric vacuum, the associated functionality must consider the role of the user and how the user will interact with the data housed in the cyberinfrastructure. A sociotechnical systems approach that takes into account the interaction between the human and the technology [16,17] is therefore appropriate because it promotes theoretical development while enabling system designers and developers to incorporate social awareness, organizational behavior, or other underrepresented domains, such as culture, which may be a critical component in system use. Many engineered system innovations fail in terms of adoption or use due to their lack of attention on human-technology interactions that are necessary and unavoidable [18,19]. An innovative cyberinfrastructure that introduces various changes to the existing practices would potentially fail if its psychosocial implications are not recognized. Moreover, in the current era, both the continuous coevolution of society and technology [20,21], especially the emergence of virtual organizations [22] that utilize telecommunication [23] or electronic-learning tools [24-26], and a surge in the amount of digital data (ie, big data) [27] create challenges for system developers in designing user-friendly, yet adaptive and sophisticated cyberinfrastructures. HRADIS considers the importance of intuitive user interfaces that are cognizant of the psychosocial and educational backgrounds of its users. It is worth noting that achieving the adaptability and sophistication with the simplicity that user-friendliness requires may necessitate more investment in the information systems design and development processes.

### Iterative Process Model as a Conceptual Framework

Multiple databases are available, but remain disparate, making it difficult, if not impossible, for the health services researcher to conduct and collaborate on innovative and rigorous research that has currency and relevance. The literature provides evidence of the importance of the design theory in focusing on the design process in artifact development [28,29]. As such, we used a design science process framework to guide the design of an artifact that aims to improve data delivery to health services researchers such that practitioners more readily benefit from the insights and findings.

Design science is an essential component in information systems research that holds promise to improve research capabilities. Through artifact creation, health services researchers have immediate access to multiple and expanding datasets, offering opportunities for comparisons previously thought cumbersome and time-consuming. This artifact can be a construct, method, model, or instantiation [30]. This paper focused on the *method* used to design and develop a data warehouse for health services researchers.

Offermann et al [31] synthesized the design research process into 3 categories: (1) problem identification, (2) solution design, and (3) evaluation. This paper reports the information systems design *problem identification and solution design* categories only.

The literature offers multiple approaches for problem identification [32,33]. Historically, interviews were conducted with relevant end users so that designers could understand the issues as the users saw them. In addition, previous studies in the literature illuminate the problems that researchers look to solve. However, more recently, one needs to only read the headlines for problem identification: a lot of data, in many different places, accumulating very quickly. Some call this *big data*, but regardless of what label it is given, health services researchers are clamoring for efficient ways to cleanse, combine, analyze, and visualize the disparate datasets for ease of analysis, collaboration, action, and publication. Doing so holds promise to analyze and visualize combinations of data to reveal information that, when put into practice, can give their organization a competitive advantage.

Although there is much literature on design engineering information systems to accept data, there is very little literature on considerations to design solutions specific to disparate health datasets, or more simply stated, a *how to* approach [5,6].

**Figure 3:**
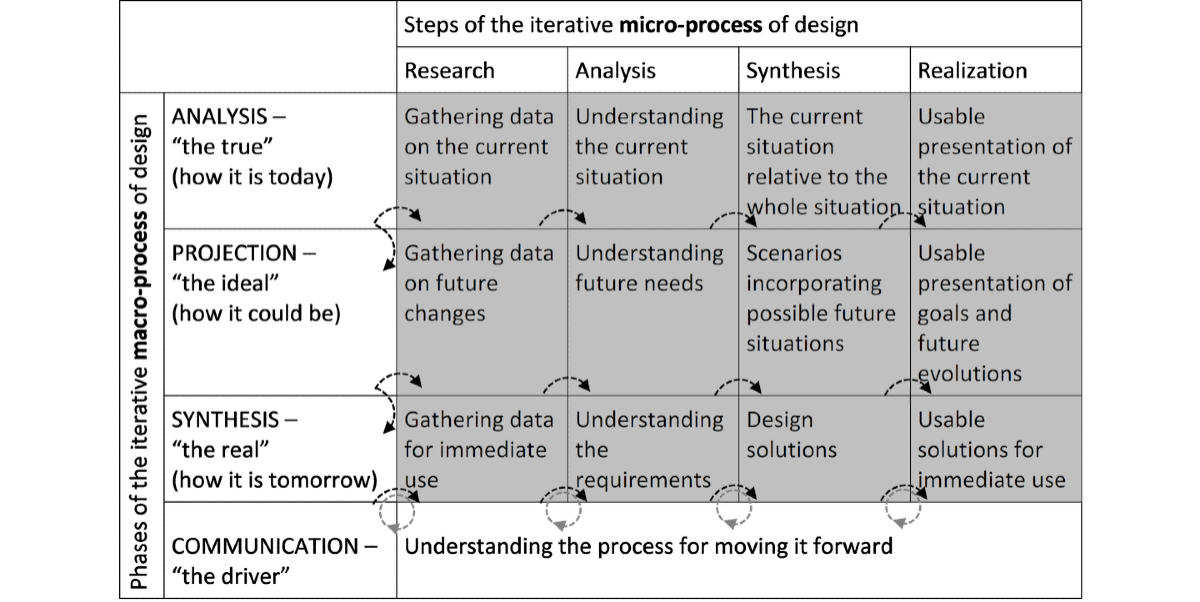
The iterative process model.

Solution design is part of an evolutionary process that helps to operationalize solutions and general system analysis and design principles. The model proposed by Jonas [34], as shown in Table 1, is appropriate for use as a conceptual model in the design and development of a data warehouse for health services researchers because it allows for consideration of the entire process and encourages creative solution design. In Figure 3, 4 domains of design inquiry (ANALYSIS, PROJECTION, SYNTHESIS, and COMMUNICATION) are indicated as *phases* of the iterative macro process of design and are denoted with all capital letters. The 4 *steps* of the iterative microprocess of design (Research, Analysis, Synthesis, and Realization), on the other hand, are *denoted with first capital and the other lower-case letters*. Each previous microprocess *step* informs the next microprocess *step*, as indicated by the arrows. Similarly, the output from each macro process *phase* of design, which considers each step of the iterative microprocess of design, then informs the next macroprocess *phase*. The dotted lines on the arrows between the microprocess steps and the macroprocess phases denote that this is an iterative process. As COMMUNICATION is the driver for all macrophases and microsteps, this continual and iterative process is denoted by a dotted circular process arrow.

The 12 shaded sections in Figure 3 contain what occurs for each respective step. For example, in the ANALYSIS/Synthesis step, there needs to be an understanding of the current situation relative to the whole. The model is not prescriptive in exactly *how* this understanding occurs and thus allows for various types of individualized design processes. Within the first 2 phases of the iterative macroprocess (ANALYSIS and PROJECTION), the model allows for moving from Research (gathering data about the problems) to Analysis (understanding those problems) to Synthesis (expressing the problems from the perspective of looking at the system as a whole and assigning the problems into categories) and then Realization (presenting these problems as a problem/requirements statement). The difference between these first 2 phases is that during ANALYSIS, the model allows us to focus on the current problems and requirements, whereas during PROJECTION, the model allows for focusing on future problems and the requirements of an ideal system. In both cases, the end product is the presentation of the problems in their respective categories. In SYNTHESIS, all the knowledge learned from the previous 2 phases informs the Research, Analysis, Synthesis (or design), and Realization (or development and implementation) of the core functionalities of the entire system, that is, the first version of the system. We address how we interpret COMMUNICATION in the *Methodology* section.

### Methodology

Consistent with the iterative process model, the first phase, ANALYSIS, is to understand the current situation with the data and then to realize a usable presentation of the current situation. This is accomplished through the following:

- Research: gathering disparate data, databases, their metadata, and the problems health services researchers currently face dealing with these data sources.
- Analysis: understanding the data, its structure, and metadata in each database and the domains of problems health services researchers face.
- Synthesis: merging these domains of problems and our understanding of the data and metadata from the perspective of the data warehouse project as a whole.
- Realization: creating an initial problem statement and requirements documentation for the project.

Table 1 presents a summary of this process relative to the ANALYSIS phase of the data warehouse project.

Once this first phase, ANALYSIS of how the data, metadata, disparate databases and their structures and evolution up to this point, and the problems around utilizing them for health services research, is appreciated, it is time to consider phase 2, PROJECTION, or what the ideal state of HRADIS would be. This second macrophase in the iterative design process is concerned with the future needs of the project. First, in the Research step, we gather data about the additional problems and requirements that may surface as a result of Analysis, Synthesis, and Realization of the ANALYSIS phase as well as data about future additions and changes of the source databases and their structures. Next, in the Analysis step, we work to understand the future needs of the ideal system based on the information gathered during the Research step. Third, in the Synthesis step, we further synthesize the future needs into possible future scenarios. Finally, in the Realization step, we present the project goal based on the anticipated needs of the data sources and system users. Table 2 presents a summary of this process relative to the PROJECTION phase of the HRADIS project.

The SYNTHESIS phase of the iterative (macro) process design considers how HRADIS will be in a usable state. First, the Research step takes into account the realizations of the previous ANALYSIS and PROJECTION phases to inform the gathering of data on the requirements for the first version of the HRADIS project that addresses its core functions with an understanding of what its future functions will be. Second, the Analysis step involves understanding the requirements of the core functions by creating process and data models for these core functions. Third, the Synthesis step involves the creation of design solutions, and finally, the Realization step involves the development and implementation of these core functionalities. Table 3 presents a summary of this process relative to the SYNTHESIS phase of the HRADIS project.

**Table 1:** The iterative process model—phase 1 (ANALYSIS).

Macroprocess	Research	Analysis	Synthesis	Realization
ANALYSIS—the true (how it is today)	Gathering data, databases, metadata, and problems researchers face using these data sources	Understanding of the data, data schemas, metadata of each data source, and domains of problems researchers face	Merging the problem domains and data/metadata analysis for the perspective of data warehouse project as a whole	Creating an initial problem statement and requirements document

**Table 2:** The iterative process model—phase 2 (PROJECTION).

Macroprocess	Research	Analysis	Synthesis	Realization
PROJECTION—the ideal (how it could be)	Gathering additional problems and requirements, including potential data sources to be added and additions and changes to the data and structure of existing data sources	Understanding future data needs and additional requirements of the ideal system	Identifying scenarios that describe user/system interaction of the ideal system from the perspective of the data warehouse project as a whole	Creating use case and project goals documents to include considerations for the future data sources and updates of the existing data sources as well as the requirements of the ideal system

**Table 3:** The iterative process model—phase 3 (SYNTHESIS).

Macroprocess	Research	Analysis	Synthesis	Realization
SYNTHESIS—the real (how it is tomorrow)	Gathering data on the requirements of the initial version of the data warehouse project that includes its core functions	Understanding of requirements of the core functionalities using process and data modeling tools	Creating design solutions based on process and data models	Development of the design solutions and implementation of the first version of the data warehouse project

The COMMUNICATION phase allows for understanding the process to move the project forward and encompasses the other 3 macroprocess phases of iterative design. The main premise of the COMMUNICATION phase is to keep the entire project team(s) on the same page as the iterative process evolves, and the design continuously changes. Considering a sociotechnical approach, COMMUNICATION also includes how the systems and its users and stakeholders interact.

As its name indicates, the model is both horizontally and vertically iterative (hence the arrows to illustrate the iterative movement); therefore, it allows for continuously updating each shaded box in Figure 3 as we increase our understanding of the requirements and the design of the project.

## Results

This section describes the application of the iterative process model to the design research process of problem identification and solution design for HRADIS.

### Iterative Process Model—Phase 1: ANALYSIS

As part of the Research step, we downloaded raw data files and data layout and/or data dictionary (metadata) files for all available data releases from the following data sources that are most frequently used by health services researchers: CMS MCR, impact/final rule files, HCAHPS, the area health resources files, AHA annual survey and IT supplement, Dartmouth Atlas, and BLS. Consistent with the iterative process model, the goal was to capture data and metadata from all of the data sources in a single database as is, without changing the source data structure. In general, data and metadata file structures for a given data source were mostly consistent among its releases. Within the release of a particular data source, there were one or multiple data files along with a metadata file. For each data file that was included in the latest release of a particular dataset, we created a table in the data warehouse, naming the table the same as the data file name with a prefix that corresponds to its data source. In cases where previous releases included a data file that was not in the latest release, we also added tables for the additional data files to the data warehouse in the same manner. For example, for the CMS MCR data source, the latest release included 3 data files, namely, ALPHA, NMRC (numeric), and RPT (report). Some of the earlier releases had another data file named ROLLUP. For each of these 4 data files, we created the following tables with an MCR prefix to indicate their data source and a HOSP prefix to distinguish them from other health organization types, for which we may include MCR data in the future: MCR_HOSP_ALPHA, MCR_HOSP_NMRC, MCR_HOSP_RPT, and MCR_HOSP_ROLLUP. Similarly, we created a table named MCR_HOSP_DATAELEMENTS for the metadata files.

Before importing data from data files into the data warehouse, we created an additional column for each table to store the release information. Then, we imported the data values from the data files into their corresponding tables, merging multiple release files into a single corresponding table. When possible, we repeated the same process for the metadata files. The aforementioned processes resulted in a database with data and metadata from all data sources, whose different releases merged into their corresponding tables with their release information preserved. Although data from different sources are not related together as an integrated database yet, having such a database allows for a better understanding of the source data structure and changes to the data structure and metadata over time and also enables data integration from different sources at the query level and the ability to save that query logic. Finally, these manual import processes inform the automation of the import tasks of future releases.

Analysis of the data, metadata, and the problems and issues the health services researchers identified helped inform the Synthesis step. In this step, we considered categories of the problems, each corresponding to a module of the system as a whole (design-focused synthesis and problem categorization). As a result, in the Realization step of the ANALYSIS phase, we generated a list of problems that HRADIS should address, as displayed in Textbox 2.

As part of the Realization step, we also developed the requirements document based on the above problem statement, as displayed in Textbox 3.

**Problem statement at the Realization step of the ANALYSIS phase.**

General problems

- Duplication of effort for each project
- Problems related to dealing with a large amount of data
- Management of licenses, data use agreements, and data access levels of users with different roles (administrative, faculty, student, etc)

Integration problems

- Disparate storage of data
- Problems related to dealing with data updates
  Updates of static data (previous release data does not change; new release data gets added to the previous releases)
  Updates of dynamic data (new releases add new data; also, update some of the previous release data)
- Integration of data elements from different data sources
  Lack of standards in how data elements from different sources are integrated
  Integration of data and metadata

Lack of standards in research data processing to deal with

- Changes of data structure from one data release to another
- Matching data elements from different releases
- Missing data values
- Inconsistent data values
- Variability and lack of documentation of assumptions about the data and the clean-up processes
- Definition and use of measures and indexes

**Requirements document at the Realization step of the ANALYSIS phase.**

The system should be able to:

- Store data and metadata from multiple data sources in a single storage (all data should be in one place)
- Store relationships among data elements within and across data sources
- Store rules and procedures for content-specific data processes
  Imputation of missing values (sometimes even multiple methods for a single data element)
  Creation of new data elements based on existing ones (calculations, indexes, conversions, etc)
  Identification of measures and dimensions
- Integrate data sources through the stored relationships, rules, and procedures
- Extract data based on predetermined criteria (data marts)

### Iterative Process Model—Phase 2: PROJECTION

Given that the PROJECTION phase deals with *the ideal* during its Research step, collaboration with fellow health services researchers is important to pinpoint potential future problems and requirements and better understand the generic workflow of a hypothetical secondary data analysis research project.

The analysis of the information gathered from the Research step provided insights into the goals and user scenarios for HRADIS. The synthesis of these insights that considers the system as a whole led to the Realization step of the PROJECTION phase, in which we identified a list of tasks and goals for the ideal data warehouse, as displayed in Textbox 4.

**List of tasks and goals for the ideal system at the Realization step of the PROJECTION phase.**

- Development of generalized solutions for
  anticipated data structure changes to the existing data sources
  addition of new data sources
- User interfaces for the system administrator user role
- In addition to provider-level data, the inclusion of patient-level data
- Addition of data sources about entities health services researchers are interested in other than hospitals (ie, nursing home data)
- Metadata search interface that allows keyword search based on a taxonomy similar to Table 4.
- A user-friendly query builder interface
- An infrastructure that allows
  multiphase larger projects (harmonious efforts)
  building new projects based on existing ones
- Inclusion of data analytics toolset
- Inclusion of data visualization toolset
- User interfaces for researchers to utilize analytics and visualization toolsets
- A knowledge base that encompasses metadata, measures and indices, analytics and visualization tools, and references related to all these knowledge base items from the literature

In addition, in the Realization step, we acknowledge the sociotechnical system theory that suggests the development of a technology by always considering the needs of end users. Relative to this project, bringing together various data sources would generate thousands of variables and measures. Moreover, sifting through thousands of variables can be very discouraging unless this process is simplified by considering the needs of the health services researchers. Therefore, to enable seamless development of research projects, an interface that allows intuitive browsing and filtering of metadata through taxonomies is a vital feature of HRADIS. Similar to biologic taxonomy, data taxonomies also separate data elements based on certain common characteristics and simplify browsing [35]. For this purpose, we have developed a data taxonomy (Table 4) by combining our own experience, information on dimensions of health care quality from the CMS Hospital Compare website [36], and hierarchical categories frequently used by health services researchers [37-42]. This taxonomy is incorporated into metadata tables and is dynamic in nature, meaning that one data element can be classified into several categories. In other words, the envisioned user interface will provide some flexibility for health services researchers in categorizing the data elements. This process embodies the use-inspired research model and facilitates further taxonomy growth and development as use and application increase.

Another product of the Realization step of the PROJECTION phase is high-level use cases for the health services researcher and system administrator user roles. Use case analysis is used in systems analysis and design to document the interaction of each user role with the system being considered to be created [43]. Use case analysis is usually performed after requirements definition and user role determination. The use cases are then used for creating the process and data models. For the health services researcher user role, use cases include browsing data elements (metadata) without creating a project, browsing completed projects and selecting one to create a new project by editing it, retrieving data for a given project, and creating a new project. Similarly, for the system administrator user role, use cases include creating system rules, editing system rules, managing user credentials, managing user groups, and managing user and group permissions.

**Figure 4:**
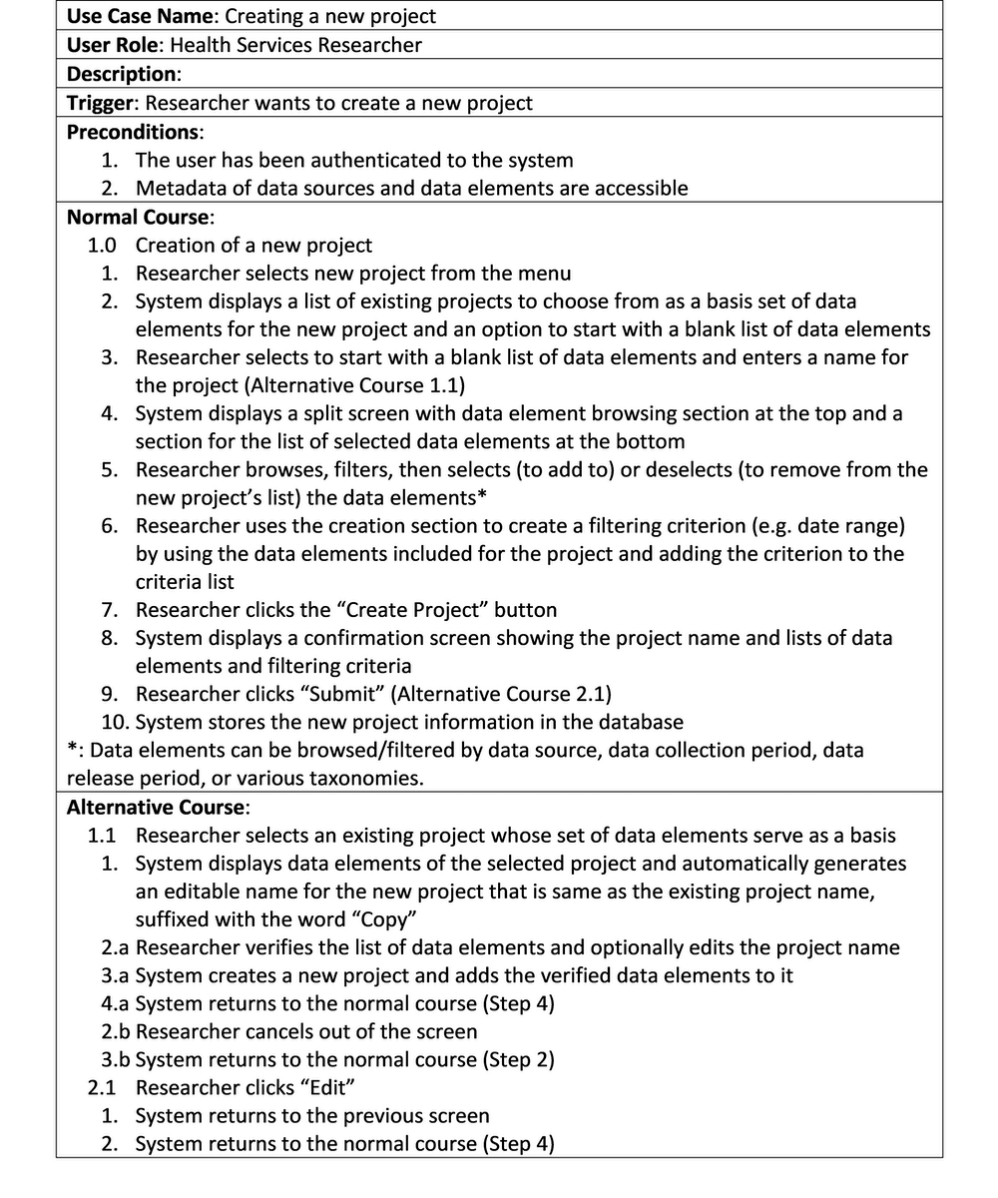
A casual format use case example.

As an example, Figure 4 shows the casual format use case for the researcher user role, which does not include the input/output data elements and their sources/destinations.

The iterative process model allows for considering *the ideal* in early stage design processes. Therefore, decision support tools facilitating the following future state use cases for the health services researcher user role are considered: (1) browsing appropriate data analytics methods, (2) selecting appropriate data analytics methods, (3) browsing data visualization methods, and (4) selecting data visualization methods.

**Table 4:** Data taxonomy for health services research.

First-level classification	Second-level classification	Examples
Organizational/structural characteristics	N/A^a^	Size (number of beds) Location System membership
Staffing	Nurse Physician Other	Registered nurse FTEs^b^ per inpatient day Physician FTEs per inpatient day Radiology technician staffing
Quality	Structural measures Patient experience Timely and effective care Outcome measures	Safe surgery checklist Communication with doctors Heart attack—aspirin at arrival 30-day readmission/mortality
Financial performance	Profitability Liquidity Capital structure Activity Utilization	Operating margin Current ratio Equity financing Total asset turnover Occupancy rate
Environmental/market characteristics	N/A	Market (ie, county, health referral region, or health service area) competition Managed care penetration Per capita income (county)

^a^N/A: not applicable.

^b^FTE: full-time equivalent.

### Iterative Process Model—Phase 3: SYNTHESIS

Considering that the SYNTHESIS phase focuses on *the real,* its Research step gathers the information from the results of the Realization steps (end products) of the ANALYSIS and PROJECTION phases to develop practical solutions for immediate use. Analysis of the initial problem statement and requirements, use cases, future requirements, and goals of the system revealed that any practical solution has to balance time spent on priority data requests for immediate research projects and time investment required for the development of the data warehouse infrastructure.

**Figure 5:**
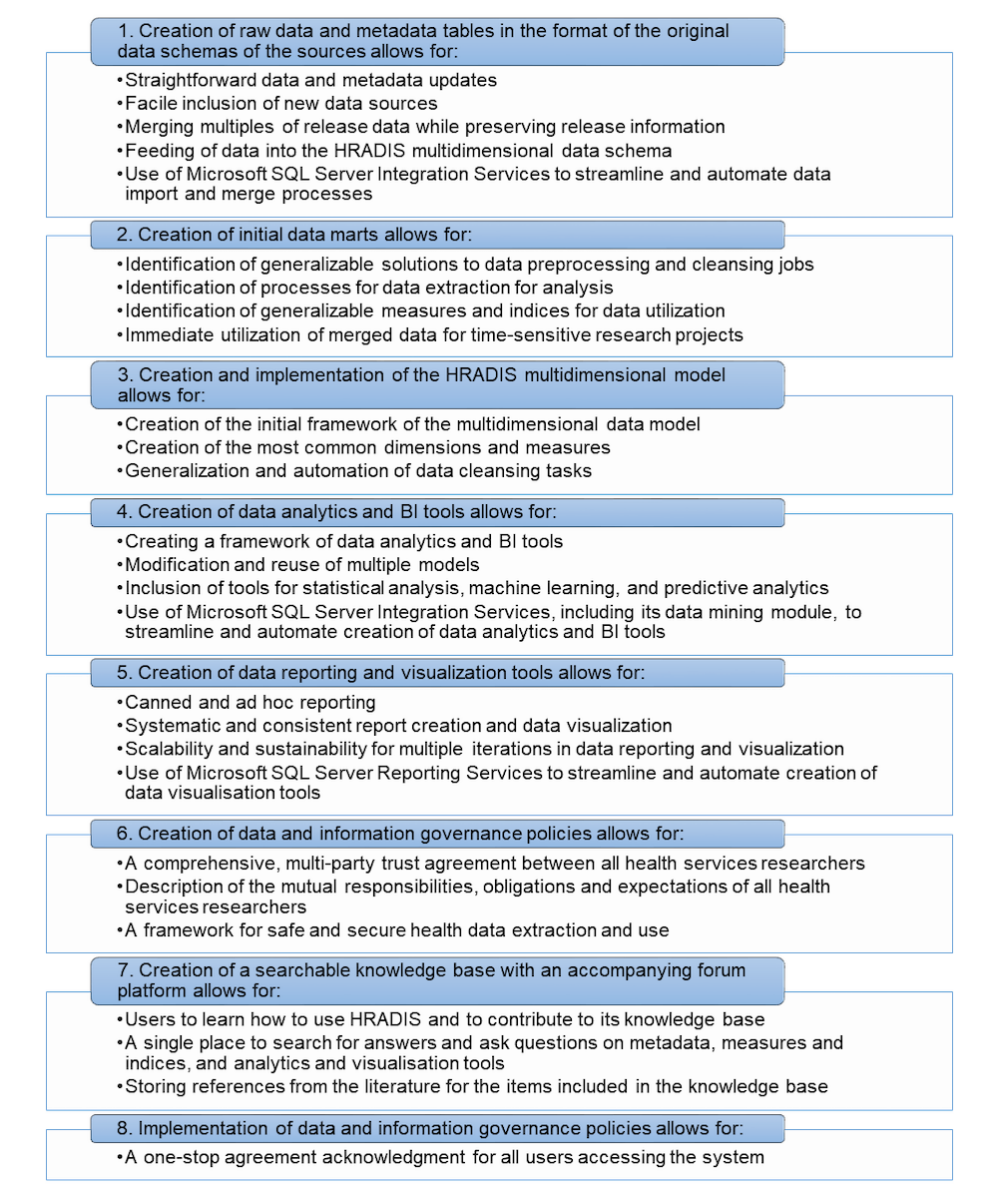
The process for a plan to implement healthcare research and analytics data infrastructure solution; BI: business intelligence. HRADIS: healthcare research and analytics data infrastructure solution.

For the Realization step, we laid out the process for a plan to implement the first version of HRADIS with its core functionalities. We then expanded this process to include high-level steps to implement some of the future functionalities, such as data analytics and visualization modules. Figure 5 explains the steps of this process, which can also be described as system modules, the reasoning behind why each module is considered, and the tasks involved in each. The iterative process model enables partial-phase completion to develop a system for immediate use, while building out other functionality in an iterative environment. As such, only various parts of the Analysis, Synthesis, and Realization steps of the SYNTHESIS phase were completed. To date, we have implemented the first 3 modules of Figure 5.

At this stage, as it was essential to obtain a better understanding of the data and the structures in the source systems, we have not yet created a multidimensional data model for HRADIS (a multidimensional model results in faster analysis and output of large and complex datasets). Instead, we kept the data schemas of the source systems in their original state, and table creation only considered the raw data and metadata tables (the first item in Figure 5). These tables will later feed data into the tables based on a new multidimensional HRADIS data schema, whose data model will be designed as part of the Synthesis step (the third item in Figure 5) and will be implemented as part of the Realization step. As part of the process, we merged data from all releases of AHA, Dartmouth Atlas, MCR, Hospital Compare, and HCAHPS data sources into their respective tables in the database. By doing so, we have encountered examples of data preprocessing and cleansing tasks from which to learn and apply in future iterations. As these examples included tasks that are typical for health services research projects, identifying generalized solutions for these assists with isolating the ETL processes, the dimensions and measures for the HRADIS multidimensional model, and the data analytics and visualization tools needed to be created as part of the next steps in the development.

## Discussion

### Principal Findings

This study provides theoretical underpinnings of the processes and methodologies in developing a data warehouse system as an infrastructure to support health services research. This paper addresses existing inefficiencies, disparate and unnecessary duplication of efforts, and the lack of harmony among health services researchers during the obtaining, extracting, preprocessing, cleansing, and filtering stages of data processing. For this purpose, we envision HRADIS as a full-service data warehouse integrating frequently used health services research data sources, processes, and methods along with a variety of data analytics and visualization tools. A conceptual iterative process model framework combined with sociotechnical systems theory provided guidance on the design process. We presented the application of 4 phases (ie, ANALYSIS, PROJECTION, SYNTHESIS, and COMMUNICATION) of the iterative process model. In the following paragraphs, the discussions on several prominent issues with supporting examples, lessons learned, reflections and recommendations, and future considerations are provided.

In terms of the application of the iterative process model (Figure 3) into the development of HRADIS, the guidance provided to synthesize the ANALYSIS and PROJECTION phases was quite significant. Traditionally, some system development strategies suggest the development and implementation of the core functionalities of a system as the first version, then additional features are added in the later phases or versions. However, the iterative process model provides a solid framework for consideration of the entire system as the pieces are being developed. With the model, the PROJECTION phase guides us to analyze the future requirements of the ideal state of the system before designing the core functionalities of the system for its first version. This allows designers to be informed by the envisioned end product of the PROJECTION phase, hence resulting in the design considerations in the SYNTHESIS phase for the *solutions for the immediate use* to include the goals of the ideal system, some of which will be designed and implemented in the future.

When a small-scale development team comprises only a few members, formal COMMUNICATION may not be as critical. In such an environment, the team is in constant communication naturally and is able to utilize agile development methods, where the features that are immediately needed are analyzed, designed, and implemented. The implemented features satisfy the immediate requirements and may later go through slight modifications to be generalized and fit into the larger project. This is also how the HRADIS project was initially implemented, by creating ad hoc data extracts, transform and load procedures, and queries for immediate research projects. Working with larger teams, on the other hand, requires more formal COMMUNICATION to create a shared understanding of the immediate processes as well as awareness of the larger *to be* project. As mentioned earlier, how the system communicates with the users and stakeholders in the general sense, and COMMUNICATION in this specific context, is critical, for example, interactions between the system and its users when there is a request for a new data source to be included in the warehouse as well as when a data extract is requested from the system.

### Conclusions

During the development of HRADIS, several issues were found that are worth further discussion. We believe that for those who consider attempting a similar project, the following lessons learned, reflections, and recommendations would be instrumental.

First, seamless progress requires a balance between immediate/urgent needs and the need to generalize the solutions being considered. To achieve this balance, we developed practical solutions by recognizing the trade-off between the quality and cost during the SYNTHESIS phase. The time investment into a highly generalized, reusable, better-quality solution to a specific problem that would yield time savings in the long term comes with its opportunity cost of not spending that particular time into multiple, less effective but working, ad hoc solutions that may yield results in the short term. For example, as we considered various geographical categorizations of hospitals based on county, health service area (HSA), and health referral region (HRR) codes in the AHA data for a study, we recognized missing values in the data and considered several ways to calculate the missing codes. We were faced with making a decision between creating a generalized solution that would encompass all possible ways to calculate the missing values or create an ad hoc solution specific to the pilot project we were working on at the time. The generalized solution would take a longer time investment to create, with the potential to be used for many studies, compared with the specific solution. In this particular case, we chose to implement the generalized solution as the long-term benefits of reuse outweighed the opportunity cost of delaying the use of HRADIS for particular pilot projects. However, these types of decisions must be considered on a case-by-case basis, as the decision would strongly depend on the potential reuse of the generalized solution and the urgency of the particular study. When making decisions on such trade-offs, one should also consider the potential benefit of the ad hoc solution in developing a generalized solution given that the ad hoc solutions sometimes provide the required knowledge base and intimacy between the designer and the data.

Second, the issue of static versus dynamic data import that was mentioned in Textbox 2 is an important consideration. When developing general solutions for data import, we realized that there was a need for two different approaches for data import processes. This need was due to inherent differences in the data sources. The former approach is static as the data source itself is static, meaning that once data are published, the content of the data does not change over time. The latter approach is dynamic as the data source itself is dynamic, meaning that the data are updated at regular intervals, and the content changes even for the archived versions (ie, years) of the data. A good example of a dynamic data source is CMS MCR; reports for earlier years can be reopened after settlement, and even the archived data are updated quarterly [44]. In our case, importing data from static data sources did not require much effort, as it was sufficient to create simple SQL scripts for import tasks. Importing data from dynamic sources requires writing SQL stored procedures that automatize and simplify the quarterly data import processes. This process addressed our ultimate goal to improve research efficiency and reduce the amount of time spent on redundant tasks.

Third, as mentioned in Textbox 3, when designing such a data warehouse, the team may consider potential ways to improve data by utilizing different data sources. In our case, sometimes, the same variable or measure existed in different datasets or was sourced from another dataset. To enhance the completeness of the data and address any missing value issues, we examined both datasets by comparing and ultimately imputing the missing values. For example, when developing certain measures, such as the Herfindahl-Hirshman index, we needed to use certain geographical market area designations such as HSA, county, or HRRs. However, due to missing information in the existing dataset for certain years, we realized that there is a need to examine the original data source (Dartmouth Atlas). Further examination revealed that the missing information could be imputed by developing an algorithm that utilizes both the information from the original data source and the existing dataset.

The fourth lesson learned pertains to the importance of the iterative design process. The conceptual iterative process model framework adapted from Jonas [34] was very useful during the development of HRADIS. Although the iterative back and forth movements may be initially perceived as inefficient and time-consuming, they were crucial in developing generalized design solutions that are beneficial in the long term. Although it may be tempting to develop a system in response to urgent data needs, we found it essential to adhere to the iterative process model. Doing so created a development expectation with our colleagues.

In the future, we plan to improve HRADIS by drawing on by the successful growth strategy and story of research electronic data capture (REDCap). Doing so considers that both HRADIS and REDCap are products of academic research and have ambitious goals, but they start small because of limited resources [45]. We plan to collaborate with researchers who have potential contributions by asking them to work with us in generalizing their contributions to fit the HRADIS framework. In this way, the contributor would have access to all the HRADIS offerings, and the existing user base would have access to the new contribution (within the data governance limitations).

## Abbreviations

AHA: American Hospital Association
BI: business intelligence
BLS: Bureau of Labor Statistics
CMS: Centers for Medicare and Medicaid Services
EHR: electronic health record
ETL: extract, transform, and load
HCAHPS: Hospital Consumer Assessment of Healthcare Providers and Systems
HRADIS: Healthcare Research and Analytics Data Infrastructure Solution
HRR: health referral region
HSA: health service area
IT: information technology
MCR: Medicare cost reports
NBER: National Bureau of Economic Research
ResDAC: Research Data Assistance Center
REDCap: research electronic data capture
WRDS: Wharton research data services
